# *EZH2* Gene Knockdown Inhibits Sheep Pituitary Cell Proliferation via Downregulating the AKT/ERK Signaling Pathway

**DOI:** 10.3390/ijms241310656

**Published:** 2023-06-26

**Authors:** Yu Cai, Peiyong Chen, Hui Xu, Shanglai Li, Bingru Zhao, Yixuan Fan, Feng Wang, Yanli Zhang

**Affiliations:** Jiangsu Livestock Embryo Engineering Laboratory, Nanjing Agricultural University, Nanjing 210095, China; 2021205019@stu.njau.edu.cn (Y.C.); 2020105038@njau.edu.cn (P.C.); 2022105004@stu.njau.edu.cn (H.X.); 2021105035@stu.njau.edu.cn (S.L.); zhaobru@163.com (B.Z.); fanyixuan@njau.edu.cn (Y.F.); caeet@njau.edu.cn (F.W.)

**Keywords:** EZH2, Hu sheep, proliferation, apoptosis, gonadotropin secretion, pituitary cells

## Abstract

Pituitary gonadotropins perform essential functions in mammalian reproduction by stimulating gametogenesis and steroidogenesis in the ovaries and testicles. EZH2 is a histone methyltransferase that inhibits proliferation and aggravates apoptosis in stem cells subjected to pathological stimuli. However, the expression and molecular mechanisms of EZH2 in pituitary cells in vitro have not been extensively studied. In this study, the relative abundances of EZH2 mRNA (*p* < 0.01) and protein (*p* < 0.05) expression were larger in the pituitary cells of Hu sheep with relatively greater fecundity (GF) compared to those with lesser fecundity (LF). Loss-of-function examinations demonstrated that *EZH2* gene knockdown led to an earlier induction of apoptosis in sheep pituitary cells (PCs). The relative abundance of *CASP3*, *CASP9*, and *BAX* was increased (*p* < 0.01), while *BCL2*’s abundance was less decreased (*p* < 0.01) in PCs where there was *EZH2* gene knockdown. Additionally, cell proliferation (*p* < 0.01) and viability (*p* < 0.01) were decreased in EZH2-knockdown sheep PCs, and the cell cycle was blocked compared to a negative control (NC). Notably, *EZH2* gene knockdown led to reduced abundances of gonadotropin subunit gene transcripts (FSHβ, *p* < 0.05) and reduced FSH release (*p* < 0.01) from PCs. *EZH2* gene knockdown led to reduced phosphorylation of AKT, ERK, and mTOR (*p* < 0.01). The results suggest that EZH2 regulates pituitary cell proliferation, apoptosis, and FSH secretion through modulation of the AKT/ERK signaling pathway, providing a foundation for further study of pituitary cell functions.

## 1. Introduction

Suboptimal fecundity continues to be a limitation to the productivity of the sheep industry. Elucidation of the regulatory mechanisms that modulates fecundity is extremely important due to associations between increased litter size and profitability for sheep producers. Hu sheep are an important breed in sheep production enterprises due to their capacity to breed throughout the year and relatively greater fecundity as compared to most breeds used for meat production in China, with an average litter size of 2.06 [1]. Animals of this breed, therefore, are an excellent choice for investigating the molecular mechanisms modulating fecundity. FecB (bone morphogenetic protein receptor-ΙB, BMPR-ΙB) is an essential protein for ovulation to occur in sheep and has also been identified as the first major protein affecting litter size in Hu sheep [2]. Even though there have been these molecular-based elucidations, the underlying mechanisms of sheep fecundity remain largely unknown.

Pituitary gonadotropins (including FSH and LH) are the key hormones regulating the fecundity of domestic animals. Pituitary FSH and LH secretion is positively regulated by the hypothalamic gonadotropin-releasing hormone (GnRH) [3], and there is negative feedback of gonadal steroids at the hypothalamus in regulating the release of GnRH and, therefore, LH and FSH from the anterior pituitary [4]. The average concentrations of peripheral plasma FSH and LH in sheep with relatively greater fertility were significantly higher than those in sheep with relatively lesser fertility [5]. Ovarian follicular development and ovulation are primarily regulated by the functions of FSH and LH. Functions in the hypothalamic–anterior pituitary–gonadal axis are, therefore, associated with prolificacy in sheep. However, in previous studies, there was a primary focus on the endocrine and physiological mechanisms in the ovarian follicles [6,7], with few studies being conducted to evaluate the effects on the pituitary gland. Recently, numerous epigenetic modifications have been identified as affecting the release of gonadotropins from the anterior pituitary. An example is how lncRNA SM2 is involved in the regulation of gonadotropin’s release from the anterior pituitary in Hu sheep through modulating the functions induced by the oar-miR-16b/TGF-β/SMAD2 signaling pathway [8]. LncRNA-m433s1 regulates the quantity of FSH secreted by inducing an increase in the relative abundance of FSHβ through functioning as a competing endogenous RNA by interacting with miR-433 in male rats’ anterior pituitary cells [9]. However, less is known about the molecular mechanism of histone methylation in pituitary functions. Therefore, for elucidating the epigenetic effects modulating the fate of pituitary cells, it is important to understand animal reproductive processes of the hypothalamic–anterior pituitary–gonadal axis.

The enhancer of zeste homolog 2 (EZH2)—the essential catalytic subunit of polycomb repressive complex 2 (PRC2)—represses targeted genes via methylation of lysine 27 of histone 3 (H3K27) [10]. The consequences of EZH2-mediated epigenetic modifications are diverse and have significant impacts on cellular processes and disease development. *EZH2* gene mutations can lead to developmental disorders such as Weaver syndrome and Coffin–Siris syndrome [11]. EZH2 expression declines with age, and its loss has been linked to age-related diseases such as cancer and neurodegenerative disorders [12]. Understanding the mechanisms underlying EZH2-mediated epigenetic regulation is essential for the development of novel therapies for various diseases. Increasing evidence indicates that EZH2 has important functions in proliferation, invasion, and angiogenesis of multiple cancerous tissues by acting as a core epigenetic regulator, with greater-than-typical abundances reported in human pituitary adenomas (PAs) [13], so it could serve as a potential diagnostic marker for invasive pituitary adenomas [14]. Moreover, EZH2 is involved in various biological processes, including differentiation, maintaining cell identity and proliferation, and stem-cell plasticity [15]. Within the mammalian immune system, EZH2 is predominantly present in proliferating cells [16]. Therefore, EZH2 has functions that result in the maintenance of ovarian cancer stem cells (CSCs) through effects on stemness and chemoresistance, with EZH2 functioning to induce CHK1 signaling to promote ovarian cancer chemoresistance [17]. Myostatin also functions as a signaling protein to Pax7, at least through EZH2, during myoblast proliferation and differentiation in sheep [18]. EZH2 suppressed the relative abundance of HMGA2, leading to suppression of the PI3K/AKT pathway in myocardial tissues of MI rats [19]. Furthermore, EZH2 has important functions in the modulation of animal reproduction. The protein encoded by the *EZH2* gene was identified as stage-specific genes of sheep germ cells, indicating there are important functions of this protein in male reproductive processes [20]. EZH2 had differential abundances in the pituitary pars tuberalis (PT) in sheep when the animals were in a long-day photoperiod (LP); therefore, EZH2 might have functions in the regulation of seasonal reproduction [21,22]. Considering essential functions in cell integrity and reproduction, EZH2 warrants further investigation regarding potential functions in sheep reproduction. Thus, it would be interesting to determine whether there are greater relative abundances of EZH2 associated with the regulation of pituitary functions and fertility. This might lead to a greater understanding of EZH2’s molecular mechanisms in the regulation of pituitary cell functions.

In the current study, we validated the signals by RNAi in sheep pituitary cells in vitro by determining the relative abundances of mRNA and protein, as well as cells’ morphological characteristics. We also evaluated cell proliferation and gonadotropin concen-trations in the media through in vitro studies with Hu sheep PCs. Furthermore, we evaluated the functions of the EZH2-ERK pathways to elucidate whether there were epigenetic effects on EZH2 in anterior pituitary cells.

## 2. Results

### 2.1. Profiles for Relative Abundance of EZH2 in the Hu Sheep Pituitary

**Figure 1:**
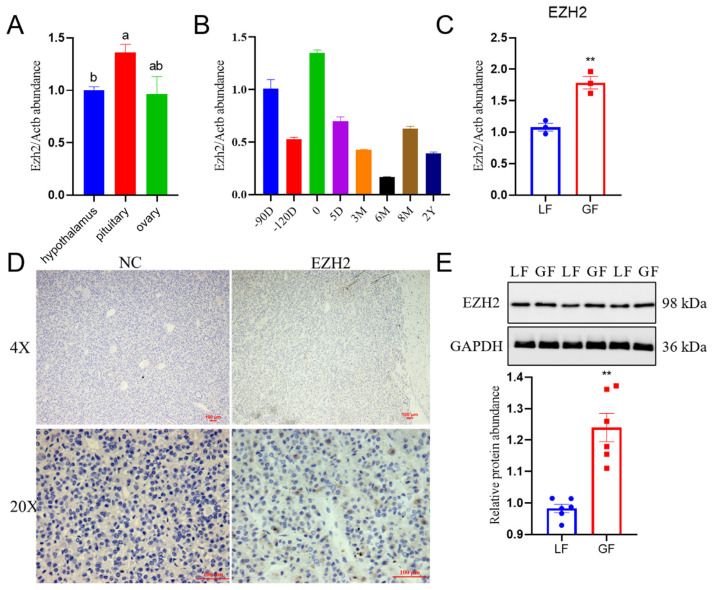
EZH2 is present in the Hu sheep pituitary cells: (**A**) Relative mRNA abundance of EZH2 was determined in the HPO axis of Hu sheep by qPCR, and β-actin was used as an internal control. (**B**) The relative abundance of EZH2, depicted at different developmental stages (90 fetus, 120 fetus, 0, 5 days, 3 months, 6 months, 8 months, and 2 years after birth) of the pituitary from Hu sheep; β-actin was used as an internal control. (**C**) EZH2 had greater relative abundance in the pituitary cells from Hu sheep with relatively greater fecundity (GF) in comparison to those with lesser fecundity (LF), and β-actin was used as an internal control. (**D**) Immunocytochemistry for EZH2 in Hu sheep pituitary cells relative to negative controls (NC). Scale bars correspond to 100 μm. (**E**) There was a greater relative abundance of EZH2 protein in the pituitary cells from Hu sheep with relatively greater fecundity (GF) relative to those with lesser fecundity (LF), as determined using Western blot procedures. Data are reported as the mean ± SEM (** *p* < 0.01). The values with different letters a and b indicate statistically significant differences (*p*-value < 0.05).

We determined the relative abundance of EZH2 mRNA transcripts in the sheep HPO (hypothalamus–pituitary–ovary) axis by utilizing qPCR procedures. There was greater relative abundance of EZH2 transcripts (*p* < 0.05) in the pituitary than the hypothalamus of Hu sheep (Figure 1A). Furthermore, there were differential abundances in the pituitary of Hu sheep at different developmental stages (90 fetus, 120 fetus, 0, 5 days, 3 months, 6 months, 8 months, and 2 years after birth), as depicted in Figure 1B. It is noteworthy that EZH2 mRNA transcripts and protein had greater (*p* < 0.01) relative abundance in the pituitary cells of Hu sheep with relatively greater fecundity (GF) compared with those with lesser fecundity (LF) (Figure 1C–E). Furthermore, there were specific cells of the anterior pituitary from Hu sheep where EZH2 was detected. Moreover, EZH2 expression was also detected in Hu sheep pituitary cells at morphological levels (Figure 1D). When there was holistic consideration of the results from the present study, there was greater abundance of EZH2 in the pituitary tissues of sheep with relatively greater fecundity than those with lesser fecundity.

### 2.2. Relative EZH2 Abundance in GnRH-Stimulated PCs

**Figure 2:**
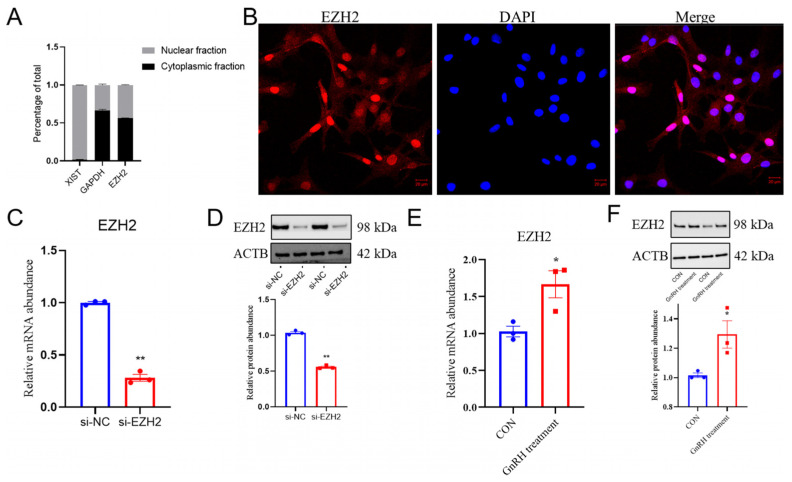
*EZH2* mRNA transcripts had greater abundance in GnRH-stimulated pituitary cells: (**A**) EZH2’s location in Hu sheep pituitary cells when using the cytoplasmic and nuclear RNA fractionation assay. (**B**) Immunofluorescence with EZH2 antibody indicating that EZH2 is located in the nucleus and cytoplasm of pituitary cells. Scale bars correspond to 20 μm. The average signal intensity was determined using ImageJ 1.42q software (http://rsb.info.nih.gov/ij, accessed on 23 June 2023). The DAPI results indicate nuclear staining. Merged results are indicative of where there were staining overlays. Suppression of the relative abundance of EZH2 in pituitary cells using si-EZH2, as confirmed using qPCR (**C**) and Western blot (**D**). Increased mRNA transcription of EZH2 was determined in GnRH-stimulated pituitary cells, as measured by qPCR (**E**) and Western blot procedures (**F**). Data are reported as the mean ± SEM (* *p* < 0.05, ** *p* < 0.01).

To determine the functions of EZH2 in gonadotropin secretion, we initially determined in vitro whether EZH2 was present in the nucleus and cytoplasm of PCs, and EZH2 was present in both cellular locations (Figure 2A). The immunofluorescence results (Figure 2B) were consistent in that EZH2 was detected in PCs using this procedure. Furthermore, when knockdown procedures were applied to the *EZH2* gene using small interfering RNA (siRNA), the EZH2 transcript and protein abundances were less than in PCs where there was no knockdown of the *EZH2* gene (*p* < 0.01) (Figure 2C,D). It is noteworthy that GnRH stimulation induced an increase in the relative abundance of EZH2 transcripts (*p* < 0.05) relative to the control group (NC), as shown in Figure 2E,F.

### 2.3. Effects of EZH2 Abundance Downregulation on Proliferation in PCs

**Figure 3:**
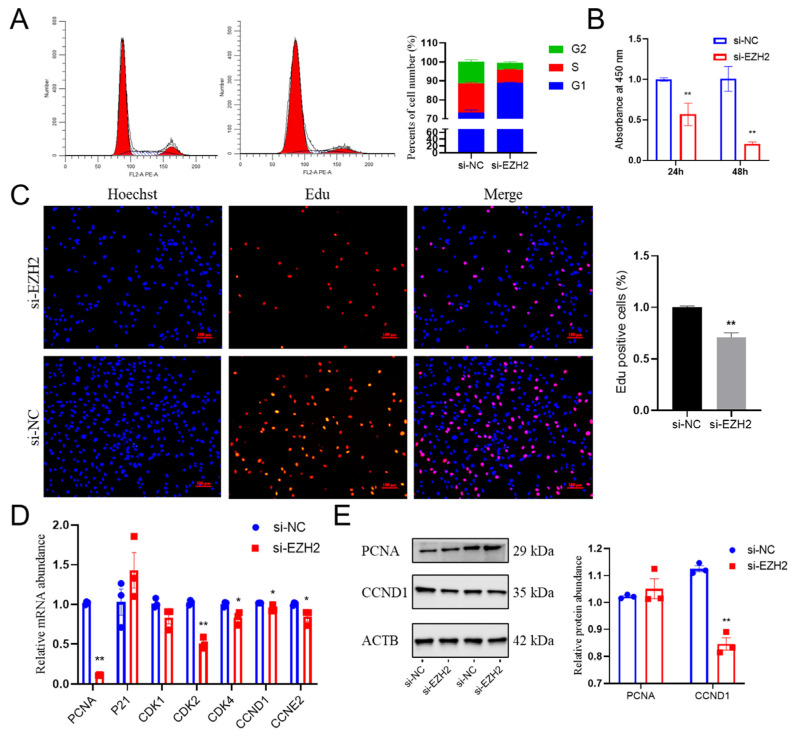
EZH2 inhibition limits the proliferation and cell cycle of PCs’ functions: (**A**) Downregulation of *EZH2* gene expression induced an arrest of the cell cycle at the S phase in PCs. Vertical axis: % of cells. (**B**) CCK-8 results indicating that the suppression of EZH2 reduced the proliferation potential of PCs. (**C**) Sheep PCs treated with siEZH2 had reduced Edu incorporation compared to the NC. Hoechst staining results indicated that EZH2 was present in the nucleus. Scale bars correspond to 100 μm. (**D**) Relative abundances of *PCNA*, *CDK2*, *CDK4*, *CCND1*, and *CCNE2* mRNA transcripts were less in EZH2-knockdown sheep PCs compared to the NC, as determined using qPCR. (**E**) The relative abundances of PCNA and CCND1 were reduced as a result of silencing of the *EZH2* gene. Data are reported as the mean ± SEM (* *p* < 0.05, ** *p* < 0.01).

Using interference efficiency procedures, we determined the importance of the functions in PCs. Flow cytometry results indicated that the population of cells was larger during the S phase of the cell cycle in the EZH2-downregulated pituitary cells (Figure 3A). The results from the 5-ethynyl-20-deoxyuridine (EdU) assay indicated that knockdown of EZH2 inhibited cell proliferation (Figure 3C), which is consistent with the results obtained using the Cell Counting Kit8 (CCK8) assay (Figure 3B). Consistent with these results, the relative abundances of several cell-cycle regulators, such as cyclin D1 (CCND1) and PCNA, was less (*p* < 0.05) than before EZH2 inhibition (Figure 3D,E). Furthermore, when there was knockdown of the *EZH2* gene expression, there was markedly less CDK2 (*p* < 0.01), CDK4, and CCNE2 (*p* < 0.05) in Hu sheep PCs as compared with cells where this knockdown did not occur. In general, these results suggest that knockdown of *EZH2* gene expression leads to suppression of the proliferation from PCs as a result of inducing cell-cycle arrest.

### 2.4. EZH2 Gene Knockdown Induces Cell Apoptosis in PCs

**Figure 4:**
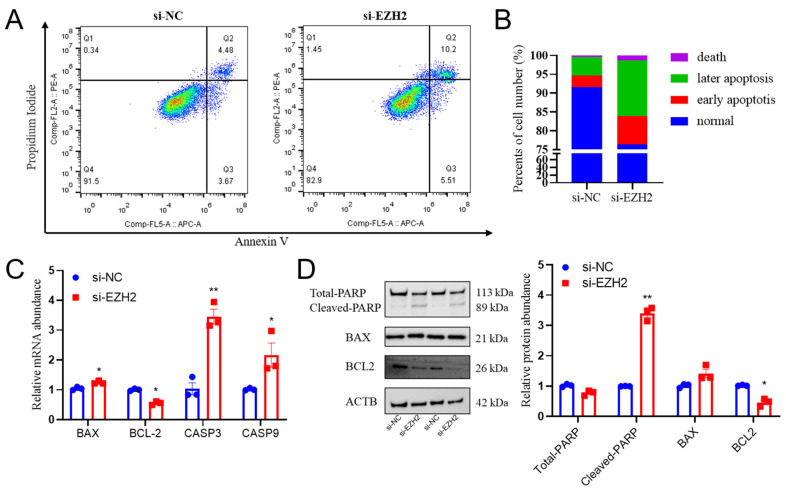
Knockdown of the *EZH2* gene led to greater apoptosis in PCs: (**A**,**B**) Downregulation of *EZH2* gene expression led to enhanced apoptosis in sheep PCs. (**C**) Relative abundances of BAX, BCL2, CASP3, and CASP9 were greater in PCs where there was downregulation of *EZH2* gene expression in comparison with PCs of the control group (NC). (**D**) Relative abundances of total PARP, cleaved PARP, BAX, and BCL2 were altered when there was downregulation of *EZH2* gene expression. Data are reported as the mean ± SEM (* *p* < 0.05, ** *p* < 0.01).

To further elucidate the physiological functions of EZH2 in sheep PCs, we investigated the effects of *EZH2* gene knockdown on the occurrence of apoptosis. The flow cytometry results indicated that *EZH2* gene knockdown induced PC apoptosis (Figure 4A,B). Furthermore, with the use of this procedure, there was reduced abundance of BCL2 (*p* < 0.05) and greater abundance of BAX (*p* < 0.05), CASP3 (*p* < 0.01), and CASP9 (*p* < 0.05) mRNA transcripts, as well as the BAX/Bcl-2 ratio in Hu sheep PCs (Figure 4C). Similarly, the relative protein abundance of cleaved PARP and BAX were larger (*p* < 0.01), but the abundance of BCL2 was less (Figure 4D), which is consistent with the results when using RT-qPCR procedures.

### 2.5. EZH2 Gene Silencing Led to Reduced FSH Secretion from PCs

**Figure 5:**
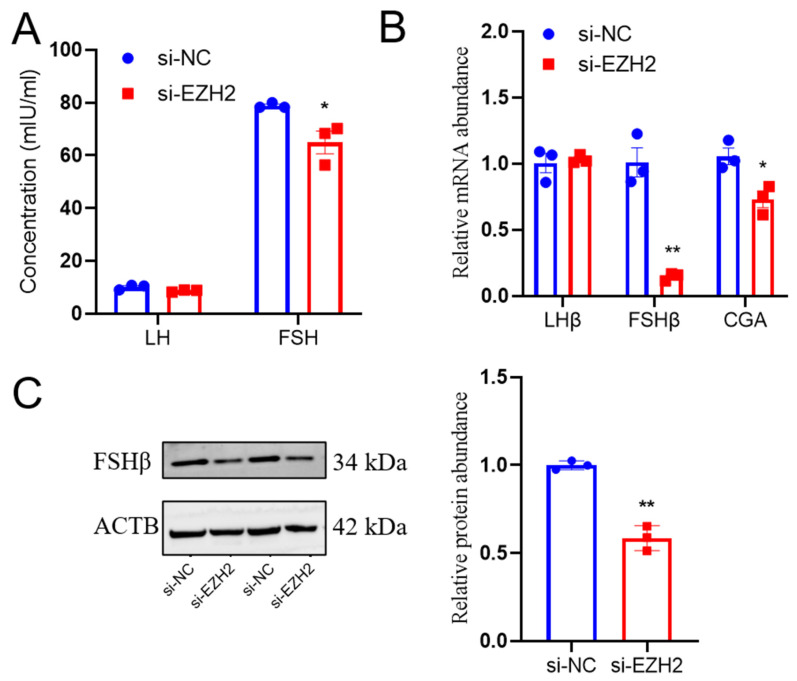
*EZH2* gene silencing led to decreased FSH secretion from PCs: (**A**) *EZH2* gene downregulation resulted in reduced FSH secretion from sheep PCs. (**B**) The relative abundance of LHβ, FSHβ, and CGA mRNA transcripts was less in PCs when there was *EZH2* gene knockdown in sheep PCs compared to the PCs of the NC group. (**C**) There was less FSHβ in PCs when there was *EZH2* gene knockdown. Data are reported as the mean ± SEM (* *p* < 0.05, ** *p* < 0.01).

*EZH2* gene knockdown led to decreased FSH secretion (*p* < 0.01) from Hu sheep PCs, but there were no effects on LH secretion (Figure 5A). Furthermore, *EZH2* gene silencing led to reduced abundance of subunits such as FSHβ mRNA transcripts (*p* < 0.01) and CGA mRNA transcripts (*p* < 0.05), but there was no effect on abundance for the LHβ mRNA transcripts compared to the NC group (Figure 5B). Similarly, there was a lesser relative abundance of FSHβ protein in PCs when there was EZH2 knockdown (Figure 5C). Overall, these results indicate that EZH2 has functions that lead to a decrease in the release of FSH from the anterior pituitary by suppressing the biosynthesis of FSHβ.

### 2.6. EZH2’s Effects on Regulating the AKT/ERK Pathway in PCs

**Figure 6:**
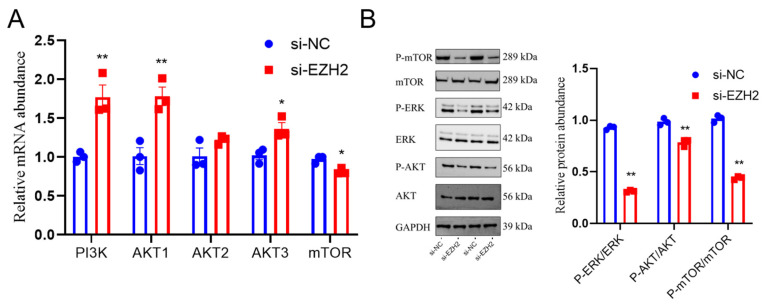
EZH2 may have functions in the AKT/ERK-mTOR pathways: (**A**) The relative abundance of PI3K, AKT1, AKT2, AKT3, and mTOR in PCs when there was *EZH2* gene silencing. (**B**) Relative abundances of ERK, AKT, and mTOR proteins and their phosphorylation in PCs of sheep where there was *EZH2* gene knockdown, compared with the NC group. Data are reported as the mean ± SEM (* *p* < 0.05, ** *p* < 0.01).

To explore the mechanisms of EZH2-regulated cell proliferation, apoptosis, and FSH secretion, there was evaluation of the AKT/ERK-pathway-related genes/proteins. *EZH2* gene deletion led to a greater relative abundance of PI3K (*p* < 0.01), AKT1 (*p* < 0.01), and AKT3 (*p* < 0.05) mRNA transcripts, whereas this deletion resulted in reduced relative abundances of mTOR (*p* < 0.01) mRNA transcripts (Figure 6A). Deletion of the *EZH2* gene led to a lesser ratio of p-AKT (Ser473)/AKT, p-mTOR (Ser2448)/mTOR, and p-ERK1/2 (Thr202/Tyr204)/ERK (*p* < 0.01) protein abundance in PCs (Figure 6B).

## 3. Discussion

EZH2 has several actions in human pituitary adenomas (PAs) [14], regulating cell proliferation and apoptosis [23], development [16], and differentiation [19]. There has not been any evaluation of EZH2 biosynthesis in food-producing animals. Therefore, in the present study, we evaluated EZH2 in the Hu sheep pituitary. Furthermore, we evaluated how EZH2 is transcriptionally regulated and whether it is involved in pituitary cell proliferation and gonadotropin secretion. Pituitary homeostasis is modulated by a balance of ERK and PI3K/AKT/mTOR signaling [24]. The results from the present study provide novel insights into the actions of EZH2 in Hu sheep pituitary cells. EZH2 knockdown led to an inhibition of cell proliferation and a reduction in FSH secretion from Hu sheep PCs in vitro by regulating the AKT/ERK signaling pathway, thereby identifying a novel molecular pathway of FSH and LH secretion in pituitary cells.

EZH2, a histone–lysine N-methyltransferase, belongs to the polycomb group (PcG) protein complex. PcG proteins modulate chromatin’s structure to repress gene transcription as an important epigenetic regulator. In the present study, there was greater relative abundance of EZH2 with a pattern of associated effects, indicating that EZH2 is an epigenetic regulator of pituitary cells. *EZH2* mRNA transcript abundance was greater in the pituitary, indicating a function in the regulation of the HPO axis. Furthermore, EZH2 mRNA transcripts had greater relative abundance in the pituitaries of Hu sheep with greater fecundity as compared to those with lesser fecundity. This indicates a possible function of EZH2 in pituitary regulation and homeostasis, which is consistent with previous findings where there were greater abundances of this transcript in pituitary adenomas [13]. Moreover, genetic and epigenetic variations were found to be involved in different types of PAs [25]. Epigenetic modifications affected the FGFR2-IIIb isoform mRNA transcript, in which has relatively lesser abundance in pituitary tumors [26]. Given the centrality of gene expression changes and the copy number alterations in pituitary tumors, epigenetic modifications have received considerable attention for further exploration [27]. EZH2-mediated epigenetic modifications have diverse consequences and play critical roles in various cellular processes, developmental disorders, cancer, and aging [11,12]. The overexpression of EZH2 leads to the silencing of tumor-suppressor genes, promoting cancer cell proliferation, invasion, and metastasis. Collectively, these reports and our results support the assumption that epigenetic mechanisms are involved in the pituitary functions.

Furthermore, the results from the present study provide new insights into the functions of EZH2 in Hu sheep pituitary cells. EZH2 knockdown led to an inhibition of cell proliferation and a reduction in gonadotropin secretion, indicating a potential action in pituitary functions. Histones have essential functions in regulating gene expression and genome imprinting, which are associated with cellular changes [28]. PcG proteins repress key genes involved in the cell cycle to indirectly regulate cell proliferation. EZH2 is essential for cell proliferation in tumor cell lines [29]. Selective inhibition of EZH2 by ZLD10A inhibits H3K27 methylation and is lethal in mutant lymphoma cells; therefore, it suppresses lymphoma cell proliferation [30]. The lesser population of Edu-positive cells and the lesser relative abundance of PCNA in PCs when there was knockdown of the *EZH2* gene are consistent with results from a previous report where there was inhibited proliferation of EZH2-deficient ESCs [31]. Cell apoptosis is regulated by proteins of the BCL2 family, the cysteinyl aspartate specific proteinase (Caspase) family—typically CASP3 and CASP9—and P53 [32]. EZH2 inhibition has effects on cell proliferation and induces apoptosis [33]. *EZH2* gene knockdown led to enhanced spermatogonia apoptosis during spermatogenesis in mice [34,35]. The results from the present study suggest that *EZH2* gene knockdown led to enhanced apoptosis in sheep PCs, as evidenced by the larger relative abundances of BAK, BAX, CASP3, CASP9, and P53 mRNA transcripts, and the lesser abundance of BCL2 mRNA transcripts, consistent with previous reports. These findings suggest that EZH2 may be a potential target for pituitary functions.

EZH2 is known to be an important regulator of the cell cycle [36], having actions in the G1/S stage by stabilizing P21, which results in decreased kinase activity and inhibited progression of the G1 phase [37]. Cyclin-dependent kinase inhibitor 1A (P21)—a downstream target of P53—inhibits the activity of the cyclin1-CDK4 and cyclinE-CDK2 complexes to inhibit progression of the cell cycle at the G1 phase and impair DNA damage [38]. Cyclin D, which interacts with CDK4, regulates the transition to the G1-S phase [39]. Cyclins D1 and E promote the proliferation of mouse SSCs and progenitor cells [40]. The results from the present study showed that the population of cells was larger during the S phase of the cell cycle in the EZH2-downregulated pituitary cells. This finding is consistent with those in other cells that have identified where there are functions of EZH2 controlling the transcription of cell-cycle regulators [31]. Ccnd3 is primarily present during the early G1 phase and is a key regulator of progression through the G0/G1 phase [41]. In the present study, the relative mRNA abundances of several cell-cycle regulators, such as CCND1, PCNA, CDK2, CDK4, and CCNE2, were markedly altered following EZH2 inhibition in Hu sheep pituitary cells, suggesting that EZH2 might affect the cell cycle. This finding is consistent with previous studies and provides further evidence of EZH2’s functions in regulating the cell cycle in sheep pituitary cells.

In previous studies, GnRH had essential functions in regulating reproduction by binding and activating GnRH receptors on pituitary gonadotropes, which synthesize and secrete gonadotropins such as LH and FSH. GnRH agonist treatment in cultured pituitary cells led to greater abundances of GNRHR and FSHβ in a transient pattern [42]. Consistently, in the present study, there were relatively greater abundances of EZH2 mRNA transcripts in GnRH-stimulated sheep pituitary cells, and suppression of *EZH2* gene expression led to reduced FSH secretion, as well as lesser relative abundance of FSHβ mRNA transcripts. These results suggest that EZH2 modulates pituitary gonadotropin secretion, and particularly FSHβ biosynthesis. Changes in gonadotrope synthesis and secretion of LH are dependent not only on changes in GnRH pulse frequency, but also on the number of GnRH receptors available for binding and, consequently, the responsiveness to a given dose of GnRH [6]. The present study detected the LH secretion of sheep PCs under normal circumstances by ELISA assay, but there was no GnRH stimulation, which showed that there were no significant effects on LH secretion and LHβ mRNA transcripts in EZH2-knockdown sheep PCs. However, the results showed a downward trend in LH secretion and LHβ mRNA transcripts, but they did not achieve a qualitative change. A possible solution would be to validate LH secretion under the GnRH stimulus in sheep PCs or in murine pituitary cells to further elucidate its function and mechanisms for further follow-up research.

There are numerous reports indicating that EZH2 is a regulator of cell proliferation and gonadotropin secretions. Although the processes through which EZH2 functions as a transcription factor have yet to be elucidated, there is evidence to suggest that multiple signaling pathways may be involved [43]. For example, EZH2 promotes OS progression via the AKT/GSK3β pathway [44], and it enhances cell proliferation and migration in trophoblast cell lines by modulating functions in the p38/MAPK signaling pathway [45]. Additionally, EZH2 has actions that induce the PI3K/AKT pathway in clinically aggressive chronic lymphocytic leukemia [46], and it modulates the cardioprotective effects of mesenchymal-stem-cell-secreted exosomes via HMGA2-mediated PI3K/AKT signaling [19]. Consistent with this finding, in the present study, knockdown of the *EZH2* gene in sheep PCs led to a decreased abundance of ERK and AKT phosphorylation, as well as the related genes’ mRNA transcripts, in a relatively consistent pattern. ERK and PI3K signaling is required to modulate pituitary homeostasis [24], which is indicative that EZH2 might promote cell proliferation through actions on the AKT/ERK signaling pathway. Considering the connection with epigenetic histone modifications and EZH2, we predicted that EZH2 might affect the biological characteristics of sheep PCs by catalyzing the methylation of H3K27. We would utilize a variety of methods for further follow-up research using a mouse model.

EZH2 is associated with cell proliferation and gonadotropin secretion by regulating the AKT/ERK signaling pathway in sheep PCs. The results from the present study indicate that there are potential functions of EZH2 in Hu sheep PCs. Further studies are needed to fully elucidate the mechanisms underlying the functions of EZH2 in pituitary regulation and diseases.

## 4. Materials and Methods

### 4.1. Animals and Sample Collection

Hu sheep (aged 2–3 years), with similar body conditions and three records of birthing the same number of lambs, were selected from Taizhou Hailun Sheep Industry Co., Ltd. (Taizhou, China) based on their pedigree records, similar to what has been reported for previous studies [8]. Ewes were assigned into relatively greater (GF, genotype FecBBB, litter size = 3) and relatively lesser fecundity groups (LF, genotype FecBB+, litter size = 1). Three ewes of each group were slaughtered within 12 h of the second natural estrus. The pituitary samples were immediately collected and stored at −80 °C for further use after lambing.

### 4.2. Cell Culture and Treatments

Hu sheep pituitaries were collected from a local abattoir (Taicang, China; 121°10′ E, 31°45′ N) during the breeding season (October to January). Pituitary tissues were dissociated using pancreatin and collagenase to isolate PCs. This was followed by isolation and identification using the relative abundance of marker genes (LHβ and FSHβ), as described in previous studies [47,48]. Briefly, PCs were seeded on different plates (6 wells, 1 × 10^6^ cells/well; 12 wells, 5 × 10^5^ cells/well; 24 wells, 1 × 10^5^ cells/well; 96 wells, 1 × 10^4^ cells/well) in DMEM/F12 supplemented with 10% fetal bovine serum (FBS) and 100 IU/mL penicillin at 37 °C in a humidified atmosphere containing 5% CO_2_. The siRNAs against EZH2, in addition to the corresponding NC, were synthesized by the Gene Pharma company (Shanghai, China); these sequences of siRNAs are listed in Table 1. Sheep PCs were transfected using 100 nM siRNAs according to Lipofectamine 3000 reagent protocols (Invitrogen Life Technologies, Carlsbad, CA, USA). Consistent with a previous study [47], GnRH powder (Ningbo Second Hormone Factory, Ningbo, China) was dissolved to 10 nM with DMEM/F12. PCs were treated with GnRH or siRNA transfection when there was 60–70% confluence, with three replicates for each treatment. The treated cells were harvested after 24 h for RNA extraction and 48 h for protein extraction.

### 4.3. Immunohistochemistry

Immunohistochemistry was performed according to previously described methods [23]. Rabbit anti-EZH2 (1:200, Abcam, Boston, MA, USA) and goat anti-rabbit immunoglobulin G (IgG) were the primary and secondary antibodies, respectively. The negative controls were incubated with bovine serum instead of primary antibodies. All sections were stained with the SABC-AP Kit (Boster, Wuhan, China) and diaminobenzidine (DAB, Boster, Wuhan, China) and examined with a light microscope (Nikon, Tokyo, Japan). All antibodies were purchased from commercial suppliers.

### 4.4. Immunofluorescence

The treated PCs were spread on round coverslips and fixed with 4% paraformaldehyde (PFA). Immunofluorescence (IF) was performed as previously described [23]. Rabbit anti-EZH2 antibody (1:200, Protein Tech, Rosemont, IL, USA) was used as the primary antibody, with Alexa Fluor goat anti-rabbit IgG (Boster, Pleasanton, CA, USA, 1:1000) as the secondary antibody, followed by incubation with 4′,6-diamidino-2-phenylindole (DAPI) for 15 min to stain the cell nuclei. Slides were examined using an LSM 710 laser scanning confocal microscope (Carl Zeiss, Oberkochen, Germany).

### 4.5. Cell Viability and Proliferation Analysis

Cell viability was evaluated using the CCK8 (KeyGen, Nanjing, China) and Alexa Fluor 555-Click-iT EDU Assay Kit (KGA332-50, Beyotime Biotechnology, Shanghai, China), as described previously [47].

### 4.6. Flow Cytometry Analysis

Transfected cells were harvested to determine the cell proliferation and apoptosis by flow cytometry analysis (Beckman Coulter Accuri C6, Brea, CA, USA). Cell apoptosis was quantified using the commercially available PE Annexin V Apoptosis Detection Kit I (No. 559763, BD Biosciences Pharmingen, CA, USA) according to the manufacturer’s instructions. After treatment, cells were digested with 0.05% trypsin without EDTA for 5 min and then washed in DPBS 3 times. Then, the supernatant was discarded, and the cells were stained with 7-amino-actinomycin and Annexin V phycoerythrin for 15 min. Finally, detection of apoptosis was carried out by flow cytometry (Beckman Coulter Accuri C6, Brea, CA, USA). Data were stored and processed using FlowJo 7.6. The first quadrant and the fourth quadrant represent the proportion of cells with early and later apoptosis, respectively, and the sum of the two is the apoptosis rate. For cell-cycle analysis, cells were harvested and then fixed overnight in 70% ethanol at −20 °C. After washing with PBS, the cells were resuspended in propidium iodide/RNase solution using PI/RNase staining buffer (BD Biosciences Pharmingen, CA, USA) for 30 min. Subsequently, the cells were analyzed e using a flow cytometer.

### 4.7. ELISA Assay

For gonadotropin-releasing hormone (GnRH)-stimulated or siRNA-transfected PCs, cell supernatant after 48 h of incubation was collected to quantify LH (Kmales biological technology Co., Ltd., Shanghai, China, DRE-S9371c) and FSH (Kmales biological technology Co., Ltd. DRE-S9373c) concentrations using an ELISA kit following the manufacturer’s instructions. The mean O.D. value for every standard and sample was calculated using a standard microplate reader (450 nm). The sensitivity of the LH and FSH ELISA assay was 0.1 IU/mL. The intra-plate and inter-plate variation coefficients were less than 15%.

### 4.8. Subcellular Localization

According to the manufacturer’s protocol, nuclear and cytoplasmic RNA were isolated from PCs using a PARIS™ kit (Thermo, DE, Waltham, MA, USA). The relative mRNA abundances were determined using qPCR. XIST and GAPDH (glyceraldehyde-3-phosphate dehydrogenase) were used as positive controls for the nucleus and cytoplasm.

### 4.9. Quantitative Real-Time Polymerase Chain Reaction (qPCR)

Total RNA extraction, reverse transcription, and qPCR were performed as previously described in [48]. All qRT-PCR reactions were performed in an ABI 7500 real-time PCR system (Applied Biosystems) using PerfectStart Green qPCR SuperMix (cat. AQ602) according to the manufacturer’s instructions. Briefly, the concentration of primers was 10 μM; the reaction components included 1 μL of cDNA template, 0.4 μL of forward and reverse primers, 10 μL of Green qPCR SuperMix, and 8.2 μL of nuclease-free water, and the total volume was 20 μL. The reaction was performed at 94 °C for 30 s, followed by 40 cycles of 94 °C for 5 s, 60 °C for 30 s, and a dissociation step consisting of 94 °C for 15 s, 60 °C for 15 s, and 94 °C for 15 s. When using qPCR for quantitative analysis, the amplification efficiency range is required to be 90–110%. The 2^−ΔΔCt^ method was used to quantify the fold change in mRNA abundances, and β-actin (ACTB) was used as the internal control. Each experiment was repeated at least three times independently. The specific primers used for qPCR are listed in Table 2.

### 4.10. Western Blot

Western blotting was performed using previously described methods [49], with slight modifications. Briefly, rabbit anti-EZH2, anti-BAX, anti-Bcl-2, anti-PCNA, and others (diluted according to Table 3) were used as the primary antibodies, while goat anti-rabbit IgG and goat anti-mouse IgG were used as the secondary antibodies. Subsequently, immunoblotting was visualized using enhanced ECL ultra-sensitive luminescence fluid (Thermo Pierce, Waltham, MA, USA) and exposed with Image Quant LAS 400 (Fiji film, Tokyo, Japan). The protein band intensity was analyzed using ImageJ 1.42q software (http://rsb.info.nih.gov/ij, accessed on 23 June 2023) (Wayne Rasband, Bethesda, MD, USA) and normalized to ACTB.

### 4.11. Data Analysis

All experiments were conducted in at least three replicates by performing biological and technical repetitions, and three independent cultures were conducted in each condition. All data are presented as mean values ± the standard error of the mean (SEM) and were normally distributed. Statistical analyses were performed using GraphPad Prism 6 (GraphPad, San Diego, CA, USA). Student’s *t*-test (two-tailed) or one-way analysis of variance (ANOVA) was performed, followed by the Student–Newman–Keuls (SNK) method for multiple comparisons. There were considered to be significant differences when there were *p*-values < 0.05 (*) and <0.01 (**), with these *p*-values being considered significant and highly significant, respectively.

## 5. Conclusions

In conclusion, the results from the present study suggest that EZH2 has stimulatory functions in pituitary gonadotropin secretion by regulating the AKT/ERK signaling pathway in sheep pituitary cells. Further studies are needed to identify potential EZH2 target genes where this protein would function as a transcription factor in regulating the pituitary functions, and to analyze the molecular biological characteristics of EZH2.

## Figures and Tables

**Table 1:** Details of siRNA sequences of EZH2 used for cell transfection.

Genes	Sense (5′–3′)	Antisense (5′–3′)
si-negative control (NC)	UUCUCCGAACGUGUCACGUTT	ACGUGACACGUUCGGAGAATT
si-EZH2-2165	GCAGCUUUCUGUUCAACUUTT	AAGUUGAACAGAAAGCUGCTT

**Table 2:** Primer sequences of genes used for reverse transcription and quantitative real-time PCR.

Gene Name	Primer Sequence (5′–3′)
EZH2-F	TGAAGAAGGGTCAGAACCGC
EZH2-R	ACATCAGACGGTGCCAACAG
FSHβ-F	GCTATTGCTACACCCGGGAC
FSHβ-R	AGTGGCTACTGGGTACGTGT
LHβ-F	CGGCTACTGCCTCAGCATGAAG
LHβ-R	CACGGGGAAGGAGACCATTGGG
PCNA-F	CCTTGGTGCAGCTAACCCTT
PCNA-R	GCCAAGGTGTCCGCATTATC
BAX-F	TTGGCTGAGTCGCTGAAGAGC
BAX-R	AACTCCCATGGCCCCCAAAT
BCL2-F	CCTTTCGTTTGCTCGTGCTC
BCL2-R	ACCTCCTCCGTGATGTGGTAT
PI3K-F	TGAAGCAATGGGTGGAGCTCA
PI3K-R	TGAGTCCTGATTCACACATAGCATCT
AKT1-F	CAGGAGGAGGAGACGATGGACTTC
AKT1-R	CCCAGCAGCTTCAGGTACTCAAAC
AKT2-F	CGACGGCTCCTTCATTGGCTATAAG
AKT2-R	AGACAGCGAATGACGAAGGTATTGG
AKT3-F	GCAGCAGCAGAGAATCCAAAC
AKT3-R	CTGCTACAGCCTGGATAGCTT
mTOR-F	CCCCAGCTGATTCCACACAT
mTOR-R	GTCTCTAGCGCGGCCTTTC
CCND1-F	ACATGGAGCTGGTCCTGGTGA
CCND1-R	GGAGGGTGGGTTGGAAATGAA
CDK1-F	CAACCTTAGAGGGGCAAGGG
CDK1-R	GGTAGCGTTGGTGAGGATCT
CDK4-F	GCTGCTGCTGGAGATGCTGAC
CDK4-R	CTCTGCGTCACCTTCTGCCTTG
ACTB-F	CGCAAGTACTCCGTGTGGAT
ACTB-R	TAACGCAGCTAACAGTCCGC
CGA-F	AAGGCCACAGTGATGGGAAA
CGA-R	CAACCATCATCAACATGGCCC

**Table 3:** Details of the specific antibodies used for Western blot procedures.

Antibodies	Cat NO.	Source	Dilution	Observed Band (kDa)
Anti-KMT6/EZH2 antibody	ab191250	Abcam (Boston, MA, USA)	1:5000	98
Anti-PCNA antibody	ab18197	Abcam (MA, USA)	1:500	29
BAX rabbit polyclonal antibody	50599-2-Ig	Protein Tech (Rosemont, IL, USA)	1:2000	24
BCL2	12789-1-AP	Protein Tech (IL, USA)	1:1000	26
AKT	60203-2-Ig	Protein Tech (IL, USA)	1:5000	56
Phospho-pan-AKT1/2/3 (Ser473)	AF0016	Affinity biosciences (Melbourne, VIC, Australia)	1:1000	56
mTOR	2983	CST	1:1000	289
P-mTOR (Ser2248)	5586	CST	1:1000	289
ERK1/2	11257-1-AP	Protein Tech (IL, USA)	1:1000	42
P-ERK1/2 (Thr202/Tyr204)	28733-1-AP	Protein Tech (IL, USA)	1:1000	42
GAPDH	60004-1-Ig	Protein Tech (IL, USA)	1:8000	36
TUBLIN	66031-1-Ig	Protein Tech (IL, USA)	1:10,000	55
β-Actin antibody	ab8227	Abcam (MA, USA)	1:4000	42
Goat anti-rabbit IgG	31460	Pierce	1:10,000	-
Goat anti-mouse IgG	20536-1-AP	Protein Tech (IL, USA)	1:2000	-

- Absent.

## Data Availability

The data that support the findings of this study are available from the corresponding author upon reasonable request.
